# Location, Location, Location: Where We Teach Primary Care Makes All the Difference

**DOI:** 10.1007/s11606-016-3966-x

**Published:** 2017-02-27

**Authors:** Christine Cassel, Michael Wilkes

**Affiliations:** 1 Kaiser Permanente School of Medicine, 1 Kaiser Plaza, 18th Floor, Oakland, CA 94612 USA; 2 University of California, Davis School of Medicine, Sacramento, CA USA

**Keywords:** primary care, medical education, population management, care delivery design, care delivery teams

## Abstract

Creating a new model to train a high-quality primary care workforce is of great interest to American health care stakeholders. There is consensus that effective educational approaches need to be combined with a rewarding work environment, emphasize a good work/life balance, and a focus on achieving meaningful outcomes that center on patients and the public. Still, significant barriers limit the numbers of clinicians interested in pursuing careers in primary care, including low earning potential, heavy medical school debt, lack of respect from physician colleagues, and enormous burdens of record keeping. To enlarge and energize the pool of primary care trainees, we look especially at changes that focus on institutions and the practice environment. Students and residents need training environments where primary care clinicians and interdisciplinary teams play a crucially important role in patient care. For a variety of reasons, many academic medical centers cannot easily meet these standards. The authors propose that a major part of primary care education and training be re-located to settings in high-performing health systems built on comprehensive integrated care models where primary care clinicians play a principle role in leadership and care delivery.

## BACKGROUND

Extensive national and international research, consistent over time, has clearly documented the importance of primary care in achieving high-functioning health care delivery and superior health outcomes.^1^^–^ 3 When primary care is readily available, health care costs are lowered, quality of care is higher, patient satisfaction improves, and unnecessary (and potentially harmful) care drops.^4^^,^ 5 Drivers of change in the USA, such as the aging population, health care reform, increased rates of chronic disease, the greater use of technology in health care, expanding roles of non-physician clinicians, and changes in consumer expectations, create an urgent need for primary care to redefine itself in ways that assure it can deliver high-quality care in this new environment. In its original form, the Affordable Care Act (ACA) accelerated payment and delivery system changes that are creating a more favorable primary care environment by improving incentives for high-quality, population-based, preventative, and comprehensive patient-centered practice.^6^ Although in the wake of the November 2016 election the ACA’s prognosis is guarded, the need for a strong primary care base as a platform for population health management will persist under almost any imaginable future.

These changes have the potential to stimulate greater primary care interest from students and residents and present an opportunity for primary care to grow and prosper.^7^ The transition from a volume-based reimbursement system to an outcomes-based system is now underway. But challenges remain. Many health care delivery systems still rely on volume-based compensation models, which often disadvantage primary care. Health information systems offer the promise of population-based management and reduced work load, but have yet to deliver on this promise. As a result, many primary physicians are frustrated rather than invigorated by the recent changes.

One of the most significant challenges to the field of internal medicine is the development of the hospital medicine specialty.^8^ While it is a generalist discipline, hospitalists do not provide the kind of comprehensive community-based care that is usually conveyed by the term “primary care.” The regular shift work and the focus on acute care and rapid patient turnover have further drained the pool of potential primary care physicians from the core generalist disciplines of internal medicine, family medicine, and pediatrics.

Other changes such as the primary care physician shortage, access issues, and the cost of care have spurred alternative approaches to delivering primary care services, including the increased use of non-physician clinicians, urgent care and retail clinics, and team approaches to the management of illness.^9^^–^ 11 Most evidence shows non-physician clinicians (NPCs) improve timeliness of care and patient satisfaction and that in low-complexity problems the quality of care is comparable between NPCs and PCPs.^5^^,^ 12 ^,^ 13 These changes will likely place primary care physicians (PCPs) in important roles managing complex patients and conditions, leaving routine and preventive care to non-physician clinicians.

Physician trainees are expected to be at the cutting edge not only of evidence-based practice, but also of new approaches to achieving the “triple aim” of delivering high-quality care, population health, and management of health care costs. Achieving these goals requires new knowledge and skills drawn from non-health related fields, such as systems engineering, that utilize data science and human factors analysis and social sciences, all in an effort to improve efficiency, reliability, and the patient expérience.^14^ Physicians and primary care practices need an infrastructure that supports these goals, which is why many predict that small practices will not survive.^15^

The education and training of primary care physicians require finding the right people (individuals), placing them in highly functioning primary care-focused training sites (institutional settings), equipping them with the right knowledge and skills, and exposing them to faculty who are positive role models and effective teachers and mentors. Trainees should be placed in learning/practice environments where the health care team is supportive and synchronized with the goal of providing patients optimal care through prevention and disease management and where systems are optimally functional. Table 1 lists problems at each of three levels—the individual, the institution, and the external environment—and within these categories a separate look at curriculum and student experiences. Much has been written about finding appropriate trainees early in their training and keeping them motivated as well as addressing the dissatisfactions of the current practice environment. A major barrier to both enticing medical students to enter the primary care fields and training tomorrow’s PCPs can be overcome by placing trainees in optimal practice environments. Therefore, recommendations focus on the location of training, which influences the faculty as well as the curriculum.

**Table 1:** Education in Primary Care

Challenge	Opportunity
I**ndividual factors**	
Students come to medical school with strong interest in primary care, which declines over time	Demonstrate positive role models, reduce burnout among teachers
Students select specialty training based on accrued debt	Provide loan forgiveness or free tuition
Burnout in medical school and resident role models has an impact on student choice of specialty	Actively address student wellness throughout medical school and build programs to develop resiliency but also address wellness of post graduates (residents) and faculty in order to model a caring balanced lifestyle
**Institutional factors**	
Reliance on older methods of learning focused on knowledge acquisition	Embrace transformative models of learning that stress active professional development, teamwork, and leadership
Poor role models/mentors	Train in settings with high physician satisfaction, effective teaching skills, and a commitment to faculty development
Primary care not respected by faculty or other residents	Train in settings where primary care is expected as essential to do patient outreach
Students (and non-primary care faculty) perceive a lack of “expert knowledge” required in primary care	Add special expertise in health and delivery science to generalist clinical knowledge. Exhibit expertise in rounds and conferences by faculty
Training not relevant to practice (tertiary care hospital vs. ambulatory)	Base curriculum in high-performing health systems roles for primary care
Failure to understand effective teamwork	Develop curriculum with an interprofessional team and have clear outcome and assessment measures for all disciplines
Little exposure to longitudinal care	Create meaningful longitudinal curriculum
Assessment exercises do not match practice	Create practice-based evaluations that include assessment of practical skills, decision-making, communication, and feedback from physicians, staff, and patients (360°)
Town/gown phenomena	Base training in clinically excellent community-based system with strong leadership
Few opportunities to train academic primary care physicians as clinician and scholar	Educators are selected and rewarded for quality of training of teaching and scholarship in delivery system science
Rapid change in medical knowledge and delivery models	Teach change management and knowledge acquisition skills
**External factors**	
Poor lifestyle—unpredictable hours, unmanageable demands	Provide learning in effective delivery systems that rely on teamwork to provide high-quality 24/7 care and a commitment to infrastructure supporting work life balance
Overwhelming effort to communicate with patients and manage/coordinate their illnesses	Combine telemedicine, electronic communications, data management, and community engagement to foster effective communication and coordination
Poor compensation relative to other specialties (strong correlation between specialty choice and compensation)	Create reimbursement system based on time and outcomes rather than procedures. Trends toward value purchasing and rewarding population health
Continuing professional development fails to address ongoing learning needs of primary care community	Promote health care delivery organizations to define their educational mission with strong linkages between practice and education. Promote visible leadership of new primary care physicians

## THE INDIVIDUAL

Many more medical students come to medical school with an interest in primary care than graduate with that same interest. The reason for this shift may be that expectations may not match reality (if the role models do not inspire and the settings do not make the physician feel effective and valued), and other areas of practice become more attractive for a variety of reasons, such as financial gain, greater perceived intellectual mastery and stimulation, more controllable lifestyle, and higher status. In contrast to other areas of the world with advanced delivery systems where primary care is highly valued, in the US fewer than 25% of medical school graduates will end up practicing primary care.^16^

In an attempt to diversify graduates, more medical schools are trying innovative approaches to the admissions process, seeking applicants with broader capabilities and attributes than can be assessed by quantitative measures, such as MCAT scores and undergraduate grade averages. It seems intuitive that students who enter school with enthusiasm for the personal relationships of a generalist practice, possess the technical and interpersonal skills to embrace systems science, and are eager to be part of a changing delivery environment will be drawn to primary care practice. But this will only happen if they see that role as exciting, rewarding, and manageable. One motivated student, who wanted to pursue primary care but saw frustrated faculty and a poorly functioning system, said to her advisor, “I admire Mother Teresa, but I don’t want to be her.”^17^ Students need to see a model where they can be good doctors without having to be saints, where they can be agents of change rather than victims of change, and where they can envision a lifestyle and practice that is personally and professional satisfying.

### The Experience of the Student

In many training programs, students learn the principles of primary care but are then placed in clinical environments where it is very difficult to implement and practice those principles. This is but one example of the “hidden curriculum”—where one thing is taught in the classroom but something quite different is observed in the practice setting. Patients are seen episodically; comprehensive information from other sources of care is not available; social services, mental health, and community resources are not easily available to address patient’s complex social and behavioral problems; and specialty care is difficult to obtain when needed. Effective primary care medicine for the twenty-first century is no longer the classic “first contact” Starfield model. Rather, primary care now requires functioning as a member of a multi-disciplinary team, proactively identifying patients who need attention, and working collaboratively to improve health and effectively manage limited resources. Part of collaborative practice requires seamless communications with patients in their home, with community-based and long-term care facilities, and with other members of the health care team. It involves being facile with technology, such as home monitoring equipment, advanced communication equipment, and information management. When students experience primary care in a clinical setting without this infrastructure, with inadequate specialty support, with no ability to follow up on the patient’s well-being, is it any surprise they feel frustrated and overwhelmed?

Across the US, there are a number of delivery systems that offer coordinated primary care including integrated systems, such as Kaiser Permanente, Geisinger, and many of the new federally qualified health centers (FQHCs) that provide comprehensive primary and specialty care. These stand in contrast to many academic medical centers where care delivery is fragmented, communication is poor, interprofessional teams are under-developed, and primary care is often not a respected specialty. The high-functioning integrated health care systems offer students rich opportunities to experience meaningful primary care roles where outcomes are defined by the health of the community served. There are examples in the public sector such as some Veterans Administration Centers and some FQHCs. In the private sector, Kaiser Permanente, the largest private integrated delivery system in the US, combines a health plan, a system of hospitals, medical offices, and other care facilities, and a multi-specialty medical group. Part of the success of Kaiser Permanente’s health care delivery model is a result of aligned payment and health care delivery incentives, salaried physicians, and a strong emphasis on system-based practices and prevention.

## THE INSTITUTION IS THE CURRICULUM

Education and training institutions (medical schools, academic health centers, and teaching hospitals) provide the curriculum, the faculty, and the clinical settings in which trainees learn. Academic health centers have mostly succeeded by responding to incentives driving high utilization of specialty services, not by investing in coordination of care, especially in post-acute and community-based settings. New incentives that emphasize quality, value, and patient experience as drivers of payment might change this, but the “tipping point” has not yet been reached. In many of the best institutions, the “primary care” fields of internal medicine and pediatrics are largely selecting for subspecialists.^18^ There are real rewards for institutions to invest in celebrity specialists and market specialty services to their communities and their donors, a message that is clear to trainees and faculty alike.

While it is difficult to predict the future, institutions engaged in primary care training should pay close attention to the skills, knowledge, and behaviors that will be required of future primary care providers. Clearly physicians in training will need a high capacity to adapt to and manage change. Medical school programs need to start early developing the leadership skills necessary to function in a team-based model of care.^19^^,^ 20 There is a need for the application of new training and assessment methods that assure trainees have well-developed communication and clinical reasoning skills and that they can be the stewards pushing for the appropriate management of limited resources.^21^ They will also need skills related to quality improvement, patient safety, improving patient satisfaction, outcomes management, and performance measurement.

Trainees in primary care need educational contact with specialists (e.g., gynecologists, otolaryngologists, orthopedists, dermatologists, etc.), but training should focus on what a primary care provider, rather than a specialist, needs to know to practice and how to effectively interface and communicate with specialists. Primary care providers need general training in unique content areas and with special populations, such as geriatrics, palliative and end of life care, chronic disease management, women’s health, LGBT health, and substance abuse, to name but a few. This training should be integrated within the curriculum rather than treated as niche areas, but this is only going to be effective if the health care system actually functions in this way. In both medical school and residency training, longitudinal experiences with both patients and faculty are essential for training and assessment, for long-term mentoring, and will create an accurate feel for many of the benefits of primary care practice. For this reason, specialized primary care tracks that allow for longitudinal training, in both medical school and in post graduate training, are ideal training opportunities. A curriculum including all of this is not enough—the clinical system needs to embody these practices and teachers need to practice with these skills.

Some academic centers can deliver this training environment; others cannot. Some are responding to the changes in payment and accountability related to patient and population outcomes; others are not moving as rapidly as nonacademic organizations, who are more nimble perhaps because they have a more focused mission of health care delivery.

Traditional academic medical centers have deeply engrained cultures valuing basic science research, grant funding, and high-profile specialty care. Few publicly celebrate their population health data, preventive care, or publicly reported quality and safety data. Faculty promotion and success are based much more on research productivity or specialty expertise than on the successes common in primary care practice or on effective, innovative teaching and mentoring. Training tomorrow’s primary care providers in integrated delivery systems (e.g., Kaiser Permanente, Geisinger, Health Partners, Mayo Clinic, and others)—where rich information systems underlie population and quality management, where delivery system science is developed and valued, where primary care providers play a central role in collaboration with non-physician clinicians, and where there are respectful and efficient relationships across teams consisting of specialist colleagues and non-physicians—would make primary care attractive and students and residents well trained. These integrated systems provide an ideal setting for medical students and residents to learn the principles of primary care and see those principles in action, as well as for residents to gain the clinical and management expertise needed.

It is also important to create a learning environment that values wellness and creates a climate of respect and work-life balance. Such places do exist, although not often within prominent academic health centers.

The stresses of medical school and residency result in declines in emotional well-being, including serious depression among 20–25% of students at some point in their medical training.^22^^,^ 23 Some of this results from the intense workload and shifts in circadian cycles, some from the pressure of intense debt, some from exposure to residents and faculty who themselves suffer symptoms of burnout, and some from outdated teaching practices forcing deadening memorization rather than active learning.

Important issues around clinician wellness and burnout cannot be ignored when considering training the next generation of PCPs. Data suggest that burnout is higher among primary care physicians, sending a negative signal to trainees. Programs that seek to train the next generation of primary care providers will need to select physician faculty who convey joy in their work and provide trainees with education around work-life balance, self-reflection, and self-improvement. This is a time of opportunity for primary care, given the changes in payment and organization of health care that values the strengths of primary care.

## THE PRACTICE ENVIRONMENT

Fee for service drives emphasis on volume of visits and procedures, and undervalues physician time spent solving complex diagnostic problems , coordinating multiple specialists, and helping patients and families make difficult decisions about their care. Payment changes are moving away from traditional fee-for-service (FFS) reimbursement and toward what is called “value-based purchasing” (VBP). The evidence is growing that effective primary care can reduce unnecessary hospitalizations and improve care coordination that leads to better outcomes. The broader application and impact of effective primary care will depend upon new approaches to education and use of information science. In addition, the combined incentives to reduce waste and demonstrate measurable quality of care are setting the stage for a growing importance of primary care in health systems, such as Accountable Care Organizations.^24^

Creating the right practice training environment is not easy. Meaningful longitudinal connections with patients, staff, and faculty are crucial to learning primary care. Technology combined with teamwork has great potential to improve communication and quality of care if used appropriately and evaluated carefully. Effective population management and teamwork achieve many of the attributes that the best of concierge medicine does—physicians have time for patients who most need them; patients have 24/7 access to information and responses to questions; and proactive information management ensures that care is personalized to each patient’s preventive and care management needs. Co-locating training programs in these high-functioning integrated systems allows for early and constant training/monitoring to assure a capable and satisfied workforce.

Health care delivery science is central to the research agenda (innovation, discovery, and dissemination) of an education and training institution that values developing primary care physicians and specialists who work with a primary care-centric approach. The authors recommend embedding primary care training in health care delivery organizations that have extensive data resources and prioritize population-based clinical research. Current information technology can provide the trainee with a dashboard of clinically useful information containing accurate, timely data about the health of the communities they serve and of the individual patients and families for whom they provide care.

One sure thing is that the trainees will practice in a future defined by these chaotic times. So, they need to feel comfortable with change, be confident in the face of uncertainty, and be both resilient and adaptable. Physicians skilled in health delivery science and trained as outlined above will be the leaders who define the best of these new and emerging models.

In December 2015, Kaiser Permanente announced its intention to create a new medical school inspired by the perception that traditional academic medical education and training do not prepare physicians to practice toward achieving the *Triple Aim* (better health care, healthier populations, and smarter spending). Since then, Geisinger announced their acquisition of the Commonwealth Medical School, with much the same intent. Such goals are important for training PCPs, but it is just as important for specialists to be trained in an environment of teamwork and care coordination where the PCP is a critically important team member.

## CONCLUSION

Training the next generation of PCPs is crucial to our health care system and will require attention to individual, institutional, and external factors. Trainees interested in primary care should look to training programs that (1) offer longitudinal curriculum provided in the context of systems producing high-quality, comprehensive population-based care, (2) have a dedicated, clinically excellent inter-disciplinary primary care faculty skilled in health systems science, (3) embody high-functioning interprofessional teamwork, and (4) offer learning experiences that allow the application of health delivery science to the primary care setting.
